# Sealed endoscopic full-thickness resection with sentinel node navigation for early gastric cancer without endoscopic submucosal dissection indication

**DOI:** 10.1055/a-2520-9882

**Published:** 2025-04-15

**Authors:** Hidekazu Kitakata, Tohru Itoh, Shinichi Kinami, Yoshiyuki Hata, Hiroaki Kunou, Tsuyoshi Mukai, Takeo Shimasaki

**Affiliations:** 1 12857 Gastroenterological Endoscopy, Kanazawa Medical University, Kahoku-gun, Japan; 2 12857 Surgical Oncology, Kanazawa Medical University, Kahoku-gun, Japan; 3 12857 Medical Research Institute, Kanazawa Medical University, Kahoku-gun, Japan

**Keywords:** Endoscopy Upper GI Tract, Endoscopic resection (ESD, EMRc, ...), Endoscopic ultrasonography, Gastric cancer, Laparoscopy

## Abstract

**Background and study aims:**

Laparoscopic and endoscopic cooperative surgery (LECS) is a beneficial procedure that enables minimal resection of the gastric wall because the tumor can be located endoscopically. However, it is not indicated for epithelial tumors because of risk of peritoneal dissemination. Therefore, we devised a new LECS technique, known as sealed endoscopic full-thickness resection (sealed EFTR), in which the serosa was sealed with a silicone sheet to prevent escape of gastric contents and tumor cells. The aims of this study were to evaluate the safety and feasibility of a newly developed procedure and to observe its long-term outcomes, including absence of local recurrence and peritoneal dissemination.

**Patients and methods:**

Approval was obtained from the Ethics Review Committee of the Japan Consortium for Advanced Surgical Endoscopy Study Group. Between December 2011 and July 2021, at Kanazawa Medical University Hospital, 16 patients with cT1 gastric cancer were enrolled in this study. Sealed EFTR was performed in patients diagnosed with negative lymph node metastasis via intraoperative sentinel node biopsy.

**Results:**

Among the 16 enrolled patients, 12 (75%) had negative sentinel node metastases, 11 of whom underwent sealed EFTR. Except for two patients who died from other causes, no instances of metastasis or recurrence were observed during the mean follow-up period of 6.5 years (range, 2–11).

**Conclusions:**

This study suggests that appropriate case selection for sentinel lymph node biopsy could allow for oncologically safe and individualized minimally invasive surgery for early gastric cancer that is ineligible for endoscopic submucosal dissection.

## Introduction

Although endoscopic submucosal dissection (ESD) is suitable for treating most intramucosal gastric cancer cases, early gastric cancer cases, including submucosal invasive (SM) gastric cancer that are ineligible for ESD, carry risk of lymph node metastasis; therefore, gastric cancer treatment guidelines have recommended gastrectomy with lymph node dissection as standard treatment 1 . However, the lymph node metastasis rate for SM gastric cancer ranges between 18% and 20% 2 3 , indicating that lymph node dissection is frequently performed even in the absence of lymph node metastasis. Extensive lymph node dissection can damage surrounding blood vessels and nerves, consequently leading to postoperative dumping syndrome, early bloating, and other diet-related complications.

Sentinel node (SN) biopsy is useful for diagnosing lymph node metastases, and the SN theory has recently been established to apply to cT1N0 early gastric cancer with a size ≤ 4 cm 4 . However, if SN metastasis is negative, lymph node dissection may be omitted, and reduction surgery may be possible.

Laparoscopic and endoscopic cooperative surgery (LECS) combines laparoscopic

gastrectomy and ESD for local resection of gastric tumors with appropriate and minimal surgical margins. This technique avoids excessive gastric wall resection and reduces incidence of postoperative diet-related complications. In addition, unlike ESD, this technique has the advantage of being able to resect the tumor reliably, avoiding risk of positive vertical margins for early gastric cancer that is difficult to dissect due to ulcer scars.

LECS with SN navigation is expected to be a function-preserving, individualized surgery that enables minimal resection while ensuring curability for early gastric cancer cases that are ineligible for ESD.

We developed a sealed endoscopic full-thickness resection (sealed EFTR) technique that covers the serosa with a silicone sheet to prevent leakage of gastric content and cancer cells 5 6 7 . This method allows full-thickness tumor resection while confirming the tumor with endoscopy. Therefore, we conducted a clinical study of sealed EFTR with SN navigation for early gastric cancer without ESD indication to evaluate the safety and feasibility of this method.

## Patients and methods

This study was conducted as a Phase 1/2 trial to demonstrate the safety and feasibility of the newly developed sealed EFTR technique. To assess safety of the method, a target sample size of 10 patients undergoing sealed EFTR was set. In addition, long-term outcomes, including presence of lymph node metastasis and peritoneal dissemination, were evaluated.

Between December 2011 and July 2021, 16 patients with cT1 gastric cancer were enrolled in this study at Kanazawa Medical University Hospital, Ishikawa, Japan.

This study obtained ethical review and approval from the Ethics Committee of the Japan Consortium for Advanced Surgical Endoscopy Study Group (approval number: 12) and our Institutional Ethics Committee (approval number: 216).

Eligibility criteria for participants included patients aged 20 to 85 years with cT1 early gastric cancer measuring ≤ 4 cm, who were considered ineligible for ESD, and who provided informed consent to participate in this study. Specifically, this included (1) mucosal cancers with ulcer scars or local recurrence, making ESD challenging; (2) differentiated mucosal cancers with ulcers and a diameter > 3 cm; (3) undifferentiated mucosal cancers with ulcers or a diameter > 2 cm; and (4) SM cancers.

For patients meeting the above criteria, we provided detailed explanations of procedure advantages, which include smaller resection areas compared with standard operations and preservation of stomach function.

Invasion depth was assessed using endoscopic ultrasound (EUS). Patients with suspected lymph node metastases on preoperative computed tomography (CT) were excluded.

Sealed EFTR was performed in patients with negative metastasis on intraoperative SN biopsy, whereas standard lymph node dissection, followed by conventional surgery, was performed in those with positive metastasis.

Postoperative complications were evaluated using the Clavian-Dindo classification. Postoperative follow-up consisted of annual endoscopic and CT examinations and blood tests including tumor markers every few months.

### Preparation

Silicone sheets, polyglycolic acid (PGA) sheets (Neoveil, Gunze Medical, Osaka, Japan), non-woven gauze, and fibrinogen-thrombin solution were initially prepared to cover the serosa. The silicone sheet was selected for its moderate strength and flexibility, enabling it to conform to the curvature of the stomach; specifically, a 0.5-mm thick sheet was used. Next, a silicone sheet was cut into a circular shape several centimeters larger than the lesion diameter to cover the incision area.

Neoveil is a non-woven fabric made of PGA and it is cut 3 to 4 cm larger than the silicone sheet. The outer section protruding from the silicone sheet serves as an adhesive margin for attachment to the serosa using fibrin glue. Various materials for gauze to attach the silicone sheet were tested and Neoveil was selected for its thinness and fine texture, enabling rapid penetration of the fibrinogen-thrombin solution to the serosa.

Furthermore, before covering the serosa with the silicone sheet and Neoveil, a thread is pre-placed in the center to create a ring for tying onto the serosa. This facilitates easy alignment and fixation during serosal coverage.

### Procedure

#### Sentinel node biopsy

The SN is identified using a combination of indocyanine green (ICG) fluorescence and radioisotope (RI) methods.

First, the tracer was prepared by mixing ICG with ^99m^ Tc-tin colloid to achieve a concentration of 50 μg/mL. It was administered in 0.5-mL aliquots at four locations in the submucosal layer around the lesion using endoscopy on the day before surgery. Any tracer that leaked into the stomach was aspirated and cleaned as much as possible to avoid interference with fluorescence observations.

Fluorescent lymph nodes (bright nodes) were identified as SN using an infrared light-observable laparoscopic system (VISERA ELITE II, Olympus Marketing, Tokyo, Japan). SNs may be challenging to recognize, particularly when located within the adipose tissue on the greater and lesser curvatures, thereby requiring careful observation.

After lymphatic basin dissection containing the SN, the lymph nodes were isolated on a back table and examined for accumulation of ^99m^ Tc-tin colloid using a gamma probe (Navigator GPS, Tyco Health Care, Mansfield, Massachusetts, United States). Lymph nodes with accumulated dye or RI were considered SN and were promptly submitted for intraoperative histological examination.

#### Mucosal and submucosal incision

**Fig. 1:**
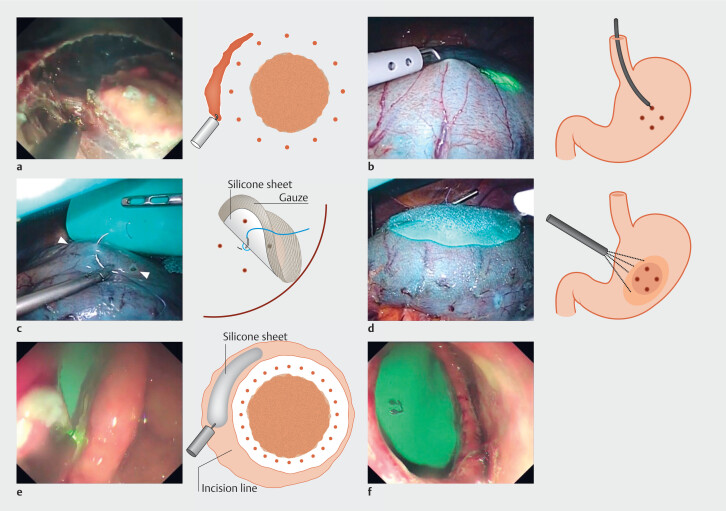
Sealed EFTR. **a** Circumference incision of the mucosa and submucosa. A deep and even incision is made until the muscular layer is exposed. **b** Marking the serosa: A high-frequency scalpel is pressed onto the mucosal/submucosal incision line, and four locations are marked on the serosa. **c** Fixing the silicone sheet. The ligation ring is ligated and fixed to the center of the silicone sheet at the center of the serosa mark. (△: Serosa side marking) **d** Serosa coating. Fibrinogen-thrombin solution is sprayed onto the PGA sheet and attached it to the serosa. **e** Incision of the serosal muscle layer. After intentionally perforating the serosal muscle layer, the tip of a high-frequency scalpel was slid over the silicone sheet to incise the serosal muscle layer. **f** Appearance after full-thickness resection. After incising the serosal muscle layer around the circumference, the thread holding the silicone sheet was cut. EFTR, endoscopic full-thickness resection, PGA, polyglycolic acid.

A circumferential incision was made in the mucosa according to the ESD procedure ( Fig. 1 **a** ). Initially, a HookKnife (Olympus Marketing) was used; however, recently, either a DualKnife (Olympus Marketing) or ORISE ProKnife (Boston Scientific, Marlborough, Massachusetts, United States) has been employed. Settings for the high-frequency generator (VIO3 or VIO300D, Erbe, Tübingen, Germany) were similar to those for ESD: mucosal incision: EndoCut, Effect 3; submucosal incision: preciseSECT, Effect 7.5 (for VIO300D, SwiftCoag, Effect 4, maximum 50 W). Local injection solution comprised epinephrine-containing saline and a small amount of indigo carmine, and its amount may be smaller than that in ESD cases. Hyaluronic acid was not used because it is believed to stagnate for a long period after surgery and cause temporary stenosis. Compared with ESD, cutting deeply until the muscle layer is exposed is crucial during submucosal incision. Some minor damage to the muscle layer is considered acceptable. The key is to make a sufficiently deep and uniform incision.

#### Serosa coverage by laparoscopic operation

Four locations were marked on the serosa side after examining the incision line with an endoscope and laparoscope ( Fig. 1 **b** ). Subsequently, the silicone sheet and Neoveil were placed using the markings as guides ( Fig. 1 **c** ). The fibrinogen-thrombin mixture was sprayed over the entire Neoveil and allowed to adhere to the serosa ( Fig. 1 **d** ).

The center of the silicone sheet and Neoveil were first ligated to paste the sheet in the appropriate position and the ligature was fixed at the center of the serosa marking.

The fibrinogen-thrombin mixture gels within a few minutes and the outer part of Neoveil adheres to the serosa. Because the silicone sheet does not adhere to the serosa, the fibrinogen-thrombin mixture is evenly spread over the entire Neoveil to prevent air leakage, assuming that a film of fibrin glue is created on the Neoveil. Finally, another layer of non-woven gauze was added and a fibrinogen-thrombin mixture was sprinkled to increase the bond strength.

#### Endoscopic serosal muscularis incision

The serosa was intentionally perforated using the DualKnife, which was subsequently inserted into the perforation. At the depth where the DualKnife sheath touched the muscle layer surface, the tip slid over the silicone sheet to incise the serosal muscle layer ( Fig. 1 **e** ). To prevent the silicone sheet from peeling off the serosa, the DualKnife was not pushed relatively hard but only moved to enable the tip to touch it lightly. Incision of the serosal muscle layer was carefully performed using the coagulation mode (preciseSECT, Effect 7.5 for VIO3; SwiftCoag, Effect 4, maximum 50 W for VIO300D).

It was removed within approximately 10 minutes. Sufficiently exposing the muscle layer is important during submucosal incision. Resection order was arranged to ensure that the upward direction of gravity was maintained at the end. This approach is necessary because attempting downward incision becomes challenging due to the weight of the tumor if it is resected from above.

#### Extraction of the resected lesion

The thread that fixed the silicone sheet was cut using the DualKnife and the resected specimen was collected through the mouth. After specimen collection, the silicone sheet and Neoveil were peeled off the serosa and pushed into the stomach. At that time, the stomach was grasped using laparoscopic forceps while ensuring no leakage of gastric juice. The stomach was temporarily opened; however, the tumor had already been removed and did not come in contact with the abdominal cavity. Finally, the silicone sheet and Neoveil were collected from the mouth.

#### Laparoscopic incision closure

The excised wound was laparoscopically closed. Suture closure was designed to minimize gastric deformation. However, it was sutured with a linear stapler if there was little deformation and was closed using hand-sewn sutures if deformation was expected or the linear stapler angle did not match. Hand-sewn sutures involved layered suturing, with continuous suturing using V-Loc (Covidien, Dublin, Ireland). After closure, the peritoneal cavity was lavaged with approximately 1 L of physiological saline and the surgery was completed.

## Results

**Fig. 2:**
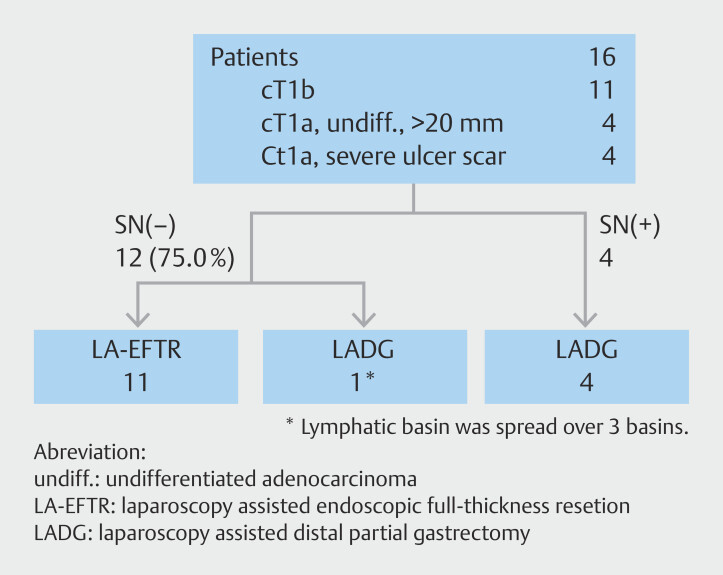
Summary of cases in which sealed EFTR was performed.

Sixteen patients with cT1 gastric cancer who were ineligible for ESD were enrolled between December 2011 and July 2021. Intraoperative SN biopsy and EFTR were performed in negative metastasis cases, whereas standard gastric resection with lymph node dissection was performed in positive metastasis cases ( Fig. 2 ).

Among the 16 patients with early gastric cancer cases, 11 were diagnosed with submucosal invasion using preoperative EUS, one was classified as M depth but ineligible for ESD due to intense ulceration, and four were undifferentiated tumors > 20 mm and considered ineligible for ESD. Twelve patients (75%) had negative SN metastases, among whom 11 underwent EFTR and one opted out of EFTR to avoid postoperative ischemia risk due to lymphatic basins extending across the three regions and instead underwent laparoscopic-assisted distal gastrectomy (LADG). Four patients with positive SN metastases underwent LADG.

Clinicopathological findings from the 11 patients who underwent EFTR were as follows: seven males and four females, with a mean age of 73.6 years (range, 59–84) ( Table 1 ). Performance status (PS) was 0 for nine patients and 1 for two patients, and there were no specific issues with performing surgery under general anesthesia. Macroscopic types were all concave, with four cases each of 0-IIa+IIc and 0-IIc, whereas tumor size was predominantly 21 to 30 mm. In addition, invasion depth was SM2 in all cases, except for those with intense ulceration in mucosal cancer and undifferentiated cancer > 20 mm, and preoperative depth diagnosis was considered accurate.

**Table:** **Table 1** Clinicopathological findings of sealed EFTR for cT1 gastric cancer without SN.

Sex			Location		
	Male	7		U	1
	Female	4		M	8
				L	2
Age					
	Mean (years)	73.6		L.C.	2
	Range (years)	59 – 84		G.C.	5
				Ant.	6
Performance status (PS)				Post.	2
	0	9			
	1	2	Histological type		
				pap	1
Macroscopic type				tub1	1
	0-IIa+IIc	4		tub2	5
	0-IIc+III	2		sig	2
	0-IIc	4		por	2
	0-III	1			
			Depth of invasion		
Tumor size (mm)				M	5*
	~20	3		SM1	0
	21~30	5		SM2	6
	31~40	3		(*: undiff. or UL1)	
			Lymphatic invasion		
				Ly0	7
				Ly1	4

Histologically, the proportion of poorly differentiated cancers was relatively high (4/11, 36.4%). This is believed to be due to the limited applicability of ESD for poorly differentiated cancers. Lymphatic invasion (Ly1) was observed in four cases (36.4%), all of which were SM2 cases.

Most tumors were located in the M region, and the anterior wall and greater curvature were common in circumference. If the tumor is located in a highly curved area, such as the lesser curvature of the gastric angle, the cardia, or the pylorus, it is difficult to apply the silicone sheet correctly. Given differences in procedure difficulty based on location, a selection bias existed regarding tumor position.

No patient had positive surgical margins and no postoperative complications, such as obstruction or early bloating, were observed. In addition, no instances of metastasis or recurrence were observed during a mean follow-up period of 6.5 years (range, 2–11), except for two patients who died from other causes.

### Case

**Fig. 3:**
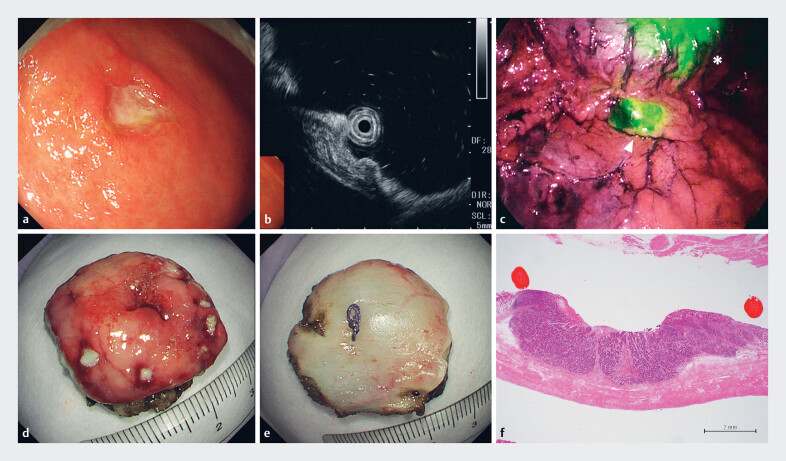
Case. **a** Preoperative endoscopic image. 0-IIa+IIc early gastric cancer, 15 mm in size, with a raised table and a deep depression found on the anterior wall of the greater curvature of the lower body. **b** EUS image: This is a hypoechoic area centered on the second layer. Thinning and irregularity of the third layer were observed. **c** Laparoscopic infrared observation. A fluorescent sentinel node is observed near the right gastroepiploic artery. (*: primary tumor, Δ: sentinel node) **d, e** Resected specimen ( **d** : mucosal side, **e** : serosal side). Excised to approximately the same size, down to the serosa, with the minimum necessary margin. **f** Histopathological findings. Submucosal infiltration is observed. EUS, endoscopic ultrasonography.

An 80-year-old male patient was diagnosed with a 15-mm 0-IIa+IIc early gastric cancer on the anterior wall of the lower body ( Fig. 3 **a** ). The lesion had a raised appearance with a deep central depression and EUS revealed thinning and irregularities in the third layer, leading to submucosal invasion diagnosis ( Fig. 3 **b** ). Preoperative contrast-enhanced CT did not reveal any lymph node or distant metastases. SN navigation-guided local full-thickness resection was performed after ensuring that the patient and family understood that this treatment was non-standard.

Using a combination of ICG fluorescence and RI methods, bright nodes were identified in regions #4d (right gastroepiploic artery basin) and #3a (left gastric artery basin) ( Fig. 3 **c** ) and the lymphatic basins were collectively dissected. Intraoperative rapid tissue diagnosis confirmed absence of lymph node metastasis and sealed EFTR was performed.

The postoperative specimen exhibited no mismatch between the mucosal and serosal surfaces and was excised with almost identical size and minimal necessary margins ( Fig. 3 **d** , Fig. 3 **e** ). Pathological results indicated tub2>tub1, T1b (SM2, 2000 μm), Ly1, V1, and N0 ( Fig. 3 **f** ).

Subsequent endoscopic examinations revealed no deformities that caused stenosis or passage obstruction, nor were there any diet-related complaints, such as leaning sensations and early satiety. The patient survived without metastasis or recurrence 7 years postoperatively.

## Discussion

In this study, 12 of 16 participants were sentinel lymph node-negative, 11 of whom underwent sealed EFTR, with no cases of metastasis or recurrence, except for two patients who died of other causes. This indicates that sentinel lymph node biopsy enabled appropriate case selection for less invasive surgery. In addition, absence of postoperative complications confirmed the safety of the procedure.

SN is a lymph node that directly receives lymph flow from a tumor, and the concept that lymph node metastases originate from the SN is termed the SN theory 4 . According to this theory, absence of metastasis in the SN indicates absence in other lymph nodes. A multicenter joint study by the Sentinel Node Navigation Surgery Study Group showed that the SN theory holds for cT1N0 early gastric cancer with a diameter ≤ 4 cm 4 . However, in gastric cancer cases, lymph nodes are small and hidden within fatty tissue, making their identification difficult. Therefore, a lymphatic basin dissection method, involving resecting the lymphatic basin containing the SN as a block and collecting the lymph nodes on a back table, was devised to reliably identify the SN 8 9 . If metastasis is negative, dissection outside the lymphatic basin can be omitted, enabling preservation of some of the gastric feeding vessels and performance of reduction surgery. A comparative study using propensity score matching in a large number of cases showed that rates of recurrence and death from other cancers were not inferior to those for standard treatment 9 .

Despite the complex lymph flow in the stomach, distribution of SN can be estimated to some extent from the tumor site 10 . The lymphatic basins are categorized into five basins along the five feeding vessels of the stomach. The main basins include the left gastric artery ( *l* -GA), right gastroepiploic artery ( *r* -GEA), and left gastroepiploic artery ( *l* -GEA). In addition, a basin may span multiple areas. For example, if the tumor is located in the greater gastric curvature at the boundary between the *l* -GEA and *r* -GEA, it may extend to the right gastric artery, *r* -GEA, and *l* -GEA. In contrast, if the basin spans all three basins, local excision is not indicated because of risk of blood flow disturbance after local excision.

In this study, 12 of 16 patients (75%) had negative SN metastasis, making it possible to accurately identify negative lymph node metastasis cases and perform reduction surgery. Although the number of cases was small and the follow-up period was short in some cases, no cases of metastasis or recurrence were observed, suggesting that individualized treatment using SN navigation was possible.

Before ESD was developed, local excision, such as laparoscopic wedge resection, was performed for early-stage gastric cancer with negative lymph nodes to preserve function 11 12 . However, accurately identifying tumor location from within the abdominal cavity is difficult, leading to complications, such as functional impairment and recurrence risk due to extended resection range and potential positive margins, respectively. Consequently, this procedure gradually ceased to be performed 12 .

As previously mentioned, LECS combines laparoscopic gastric resection and ESD for local resection of gastric submucosal tumors with appropriate, minimal surgical resection margins 13 . It has become widely used, particularly for submucosal tumors that grow in the lumen, because it prevents excessive resection of the gastric wall and deformation and stenosis of the stomach. However, it is not indicated for epithelial tumors or gastrointestinal stromal tumors (GISTs) with delle because risk of tumor deviation into the abdominal cavity and dissemination cannot be ruled out.

Therefore, to apply the LECS technique to epithelial tumors or GIST with delles, it is necessary to perform a procedure such that it does not cause peritoneal dissemination.

Thoroughly following the basic surgical procedures of oncology, such as removing the tumor to prevent its contact with the peritoneal cavity and avoiding touching the peritoneum or other organs with instruments used to grasp the tumor, is important to prevent iatrogenic peritoneal dissemination. Peritoneal dissemination has been reported to occur when the tumor dislodges into the peritoneal cavity or when the peritoneum is touched with forceps while grasping the tumor 14 . Therefore, avoiding such inappropriate surgeries is necessary.

In addition, peritoneal dissemination reportedly occurred through the omentum that adhered to the perforation site in cases where the tumor could not be completely removed due to perforation and surgery was performed several months later with the tumor left in place 15 . When the perforation is closed endoscopically with clips, the margin is everted, which may expose the residual tumor and cause contact with the surrounding organs. In both cases, contact is believed to be the cause, and procedures must be performed to avoid contact between the tumor and the surrounding organs.

Ikehara et al. 16 conducted a long-term prognostic study of perforation cases during endoscopic mucosal resection or ESD and found that none of the 90 perforation cases had recurrence of peritoneal dissemination. Peritoneal dissemination can be prevented by implementing appropriate procedures to avoid tumor contact with the peritoneal cavity. Furthermore, Huh et al. 17 did not observe peritoneal dissemination recurrence in 34 perforation cases and stated that no difference was found in long-term prognosis between cases with and without perforation.

However, risk of peritoneal dissemination due to leakage of gastric contents containing free cancer cells has also been suggested 18 19 . Hirao et al. 18 reported peritoneal dissemination recurrence in two of 22 patients who underwent ESD perforation. Both patients had early gastric cancer in the U region; although the perforation was closed endoscopically with clips, acute peritonitis developed after ESD and emergency surgery was performed. CT scans revealed ascites accumulation and intraoperative cytology of the ascites was positive. Although the time required from re-perforation to surgery is unknown, peritoneal dissemination risk due to leakage of free cancer cells is suspected.

The combination of laparoscopic and endoscopic approaches to neoplasia with a non-exposure technique (CLEAN-NET) 20 and non-exposed endoscopic wall inversion surgery (NEWS) 21 are considered techniques for removing the tumor while avoiding exposure to the abdominal cavity. In these procedures, lesion location was confirmed using an endoscope, markings were made on the serosal side, and an incision was made from the serosal side using a laparoscopic high-frequency scalpel. However, because the incision was made from the serosal side, the tumor location cannot be confirmed during the incision. The mucosal and serosal surfaces are reportedly misaligned, and the serosa may not be incised correctly 22 .

EFTR, in which the tumor is resected while confirming its location through the gastric cavity using an endoscope, is suitable to accurately and appropriately remove the tumor 23 . However, when the stomach is incised, air inside the stomach leaks, resulting in stomach collapse and a poor endoscopic view. In addition, measures must be taken to prevent infection due to gastric juice leakage and intraperitoneal dissemination due to tumor deviation. Therefore, we devised a new EFTR technique in which the serosa is sealed using a silicone sheet to maintain the endoscopic view and prevent leakage of gastric contents 5 6 7 .

Although the number of cases was small, no cases of metastasis or recurrence were observed. This method can be considered a technique that prevents peritoneal dissemination and enables full-thickness resection while confirming the tumor location. The stomach was temporarily opened when collecting the sheet after tumor resection; however, because the tumor had already been resected, it did not come in contact with the abdominal cavity.

In addition, the silicone sheet used to cover the serosa was pushed into the stomach and recovered through the mouth to avoid contact with the abdominal cavity, thereby ensuring safety by following the basic surgical procedures of oncology.

The excised wound was laparoscopically closed. Laparoscopic suturing is more established, with a higher safety and reliability. Although endoscopic suturing devices such as SutuArt have been developed 24 , current endoscopic suturing devices are technically complex and time-consuming, so we chose laparoscopic suturing for wound closure. Future advances in endoscopic suturing may make it feasible, but further refinement is required.

Closed LECS involves suturing the serosa and resecting the tumor from within the gastric lumen 25 . Similar to our method, it has the advantage of allowing precise resection while confirming the tumor position. However, Kikuchi et al. have noted that suturing the serosa causes distortion along the incision line, making it difficult to determine the appropriate dissection line, because the endoscopic view differs from that of standard ESD 25 . They addressed this problem by inserting spacers. Our method offers the advantage of enabling resection with the same endoscopic view as ESD.

Our method also has limitations regarding tumor location. If the tumor is located in the lesser curvature of the gastric angle, the cardia, or the pylorus, the silicone sheet cannot be applied correctly, and therefore, the method cannot be used.

Conditions for applying LECS to early gastric cancer are: (1) negative lymph node metastasis; (2) the tumor did not come in contact with the abdominal cavity; and (3) the tumor location could be accurately recognized and removed in the right amount. Notably, SN navigation plus sealed EFTR satisfies these conditions. Other LECS-related procedures, such as NEWS, CLEAN-NET, and closed LECS, are techniques similarly developed to prevent tumors from escaping into the peritoneal cavity; when combined with SN navigation, they may become a treatment method for early gastric cancer that is ineligible for ESD.

To date, LECS has been performed in cases in which ESD is challenging due to ulcer scars or tumor size, and for cases in which standard treatment is difficult due to advanced age or poor surgical tolerance 22 23 26 27 . Although local excision has been reported in some instances, the number of cases remains limited and the safety of LECS for gastric cancer

## Conclusions

This study suggests that appropriate case selection by sentinel lymph node biopsy could allow for oncologically safe and individualized minimally invasive surgery. Sealed EFTR combined with SN navigation may serve as a complementary treatment option for early gastric cancer alongside ESD and gastrectomy.

Furthermore, we believe that other LECS techniques, such as NEWS, CLEAN-NET, and closed LECS, could also prove to be valuable in similar contexts.

Nevertheless, this study is a report on a small number of cases and further accumulation of cases and longer observation periods will be necessary to confirm the efficacy of LECS for early gastric cancer. We anticipate future studies to provide validation.
